# Comparing Maternal Services Utilization and Expense Reimbursement before and after the Adjustment of the New Rural Cooperative Medical Scheme Policy in Rural China

**DOI:** 10.1371/journal.pone.0158473

**Published:** 2016-07-07

**Authors:** Hua You, Hai Gu, Weiqing Ning, Hua Zhou, Hengjin Dong

**Affiliations:** 1 Department of Social Medicine and Health Education, School of Public Health, Nanjing Medical University, Nanjing, China; 2 Center for Health Management and Care Security Policy Research, School of Government, Nanjing University, Nanjing, China; 3 Department of Women Health Care, the First Affiliated Hospital of Nanjing Medical University, Nanjing, China; 4 Department of Child Health Care, Maternal and Child Health Hospital of Changzhou, Changzhou, Jiangsu, China; 5 Center for Health Policy Studies, School of Medicine, Zhejiang University, Hangzhou, China; Old Dominion University, UNITED STATES

## Abstract

**Background:**

The New Rural Cooperative Medical Scheme (NCMS) includes a maternal care benefits package that is associated with increasing maternal health services. The local compensation policies have been frequently adjusted in recent years. This study examined the association between the NCMS maternal-services policy adjustment and expense reimbursement in Yuyao, China.

**Methods:**

Two household surveys were conducted in Yuyao in 2008 and 2011 (before and after the NCMS policy adjustment, respectively). Local women (N = 154) who had delivery history in the past three years were recruited. A questionnaire was used to collect information about delivery history, maternal health services utilization (prenatal care, postnatal care, and the grade of delivery institutions), NCMS participation, and reimbursement status. Logistic regression analyses were used to predict the association between policy adjustment and maternal health utilization and the association between policy adjustment and out-of-pocket proportion. Next, t-tests and covariance analyses adjusting for household income were used to compare the out-of-pocket proportion between 2008 and 2011.

**Results:**

Results revealed that compensation policy adjustment was associated with an increase in postnatal visits (adjusted OR = 3.32, *p* = 0.009) and the use of second level or above institutions for delivery (adjusted OR = 2.32, *p* = 0.03) among participants. In 2008, only 9.1% of pregnant women received reimbursement from the NCMS; however, this rate increased to 36.8% in 2011. After policy adjustment, there were no significant changes in the proportion of out-of-pocket expenses shared in delivery fee (*F* = 0.24, *p* = 0.63) and in household income (*F* = 0.46, *p* = 0.50).

**Conclusions:**

Financial compensation increase improved maternal health services utilization; however, this effect was limited. Although the reimbursement rate was raised, the out-of-pocket proportion was not significant changed; therefore, the compensation design scheme must be adjusted in practice.

## Introduction

It is well understood that maternal healthcare is the primary strategy to reduce maternal and infant mortality [1, 2]. In China, Zhejiang is a province where the maternal mortality rate and the infant mortality rate are relatively low. Zhejiang is located in China's eastern coastal economic-developed region, and the maternal healthcare utilization rate in this area is higher than in the western region. Data from the third National Health Services Survey (NHSS) revealed that all indices of maternal healthcare in Zhejiang conformed to the relevant regulations of the Ministry of Health and met the standards of women healthcare services [3]. However, there still exist some issues, especially in rural areas, such as an underuse of maternal care including systematic management, prenatal examinations, postpartum visits, and additional services [4].

Maternal and child health services utilization is influenced by many social-economic factors [5, 6]. Financial difficulty is typically among the major barriers to maternal healthcare utilization among the poor, rural Chinese population [7, 8]. To solve this problem, the Chinese government is devoted to financing health reforms and the NCMS is one of the major initiatives in rural areas.

Since 2003, the NCMS has been implemented to provide financial risk protection to the rural population. The NCMS is financed by individual contributions and by the central and local government. The NCMS operates by voluntary enrollment; it is administered at county level, although the rules and policy guidelines are determined by the central government [7]. The NCMS has a maternal healthcare benefits package. Its design and implementation vary across counties. The package usually provides reimbursement as either a fixed amount or a fixed proportion of expenditures [9]. For several years, the participation rate has consistently increased, reaching 97.5% in 2011 [10].

Previous studies have revealed that the NCMS effectively improved maternal healthcare utilization [11, 12]; however, others reported that the NCMS played a limited role or had no effect [13]. Sun’s study found that NCMS could protect farmers from the economic risks caused by diseases; however, the extent of this effect depended on scheme design and the level of financing [14]. Therefore, some local compensation schemes of NCMS have been in frequent adjustment.

Moreover, the compensation scheme adjustment differs across counties. In Yuyao, a county in Zhejiang, the compensation policy was gradually adjusted from 2008 to 2011. The outpatient compensation proportion increased from 20% to 30%, and the top line increased from 500 Yuan ($76.3) to 1000 Yuan ($152.5). Moreover, the inpatient compensation proportion also improved by 10% at each institution level, and the top line increased from 30000 Yuan ($4,575.6) to 80000 Yuan ($12,202.4). The deductible of hospitalization expenses decreased from 500 Yuan ($76.3), 1000 Yuan ($152.5), and 1500 ($228.8) Yuan to 300 Yuan ($45.8), 600 Yuan ($91.5) and 900 Yuan ($137.3), in level 1, 2, and 3 hospitals respectively.

This study used rural health-services survey data from Yuyao and conducted the analyses before and after the compensation scheme adjustment. We aimed to understand the relationship between NCMS, especially compensation scheme adjustment, and maternal healthcare utilization.

## Methods

### Study sites

This study was performed in Zhejiang province, China, which comprises approximately 55 million residents (in 2014). The economic development in Zhejiang is among the most successful of all the provinces in China. The surveys were conducted in Yuyao, which is located on the northeast of Zhejiang province. Yuyao (in 2015) has 6 streets, 15 towns, an area of 1,527 km^2^, and a population of approximately 840,000. In 2015, calculated on the registered population, the city's per capita GDP was 100,367 RMB ($16,115 at an average annual exchange rate).

### Sampling

The sample and sampling methods we used were the same as those used by the NHSS in 2008 since Yuyao was selected as a sample county in the original 2008 survey. The survey was conducted a second time in 2011 in Yuyao with the same questionnaire from 2008. A multi-stage stratified cluster random-sampling technique was used to choose the sample in both years (before and after the compensation policy adjustment). Five towns from Yuyao were drawn randomly; then, two villages were randomly drawn from each town again. Finally, the number of the households drawn from each village was determined according to the proportion of the number of households [15]. Nine-hundred households were enrolled in this survey. For our objective, only the data from 154 women who had delivered a baby prior to the survey and participated in the NCMS were examined. Half of the cases (n = 77) were investigated in 2008 and these cases delivered babies during 2006 to 2008, the other half (n = 77) were investigated in 2011 and delivered babies after 2008.

### Data collection

For this research, a leading group was established and a specific project office was utilized at the county’s Center for Diseases Prevention and Control. The office workers were in charge of training the survey instructors and investigators, organizing the investigation, and performing quality control under the supervision of Zhejiang University. The household surveys were conducted between June and August 2008 and June and August 2011, respectively. The investigators responsible for the household survey were local medical personnel (doctors from community health services centers) who were trained by researchers from Zhejiang University. After providing informed consent, all household members completed the survey at home one-by-one and face-to-face with a trained and qualified investigator. The participants provided oral informed consent. Oral informed consent in China is traditional practice. In addition, written consent was not required because no human tissue samples were utilized in this study. Ethical approval including the consent procedure was obtained from the Faculty of Public Health Ethics Committee of Zhejiang University.

The questionnaire included general household information, individual socio-economic information, individual health conditions and health services, information concerning married women of childbearing age (15–49 years old), information concerning children under five-years-old, information concerning elderly persons (aged 60 years and over), and information about diseases.

For the purposes of this study, we only examined the information from married women who had experienced childbirth the last three years to determine the impact of the NCMS reimbursement policy-adjustment on maternal services users. Our analyses consisted of the socio-demographic characteristics of the female respondents (i.e., age, education, employment, and household income), the maternal healthcare status of their recent pregnancy (i.e., prenatal care, postnatal care, delivery institution, delivery way, and parity), and their delivery fee and reimbursement status.

### Statistical analysis

The determinants of maternal health services use were analyzed using logistic regression analyses. The variables of maternal services utilization set as key dependant variables included prenatal care (≥ 5 times), postnatal care, and delivery in first or secondary institutions(According to the "standard of hospital classification management", on the basis of hospital function, facilities, technology and other indicators, all hospitals are divided into three levels). Based on study objective, a literature review, and previous practice, the key independent variables in the analyses were socio-demographics factors, parity, and the year before/after policy adjustment. Multivariate Odds Ratios (ORm) and 95% Confidence Intervals (CI) were calculated, and enter method was used for the logistic regression analyses.

To evaluate the association between NCMS scheme adjustment and reimbursement rate, the logistic regression models were fit to the reimbursement outcome. First, the unadjusted reimbursement OR for mothers recruited in 2008 versus mothers recruited in 2011 was calculated. Then, univariate OR (ORu) and 95% CI were calculated. Then, two multivariate logistic regression models were fit, the first model with only socio-demographic factors, and the second model including factors of maternal healthcare status. The dependant variable was if the delivery cost were compensated from NCMS or not. ORm and 95% CI were calculated. Enter method was selected for the logistic regression.

T-tests were used to compare the out-of-pocket (OOP) proportion between mothers recruited before and after NCMS scheme adjustment. In addition, the group differences in OOP proportion were tested using covariance analyses adjusted for income.

In the analyses, the significance level was set at 0.05. Double data entry was performed and data analyses were conducted using SAS version 9.2 (SAS Institute Inc, Cary, NC, USA).

## Results

### Participants’ general information

In 2008, the average age of surveyed women was 28.7±4.5 years old. The annual household income varied from 12,000 Yuan ($1,830.7) to 450,000 Yuan ($68,649.9) with a median of 50,000 Yuan ($7,627.8). Among the respondents recruited from 2011, the average age was 28.9 ± 4.8 years old, and annual household income varied from 10,000 Yuan ($1,525.6) to 500,000 Yuan ($76,277.7) with a median of 70,000 Yuan ($10,678.9).

Data in 2008 revealed 89.6% accepted prenatal care 5 times or more and 63.6% accepted postnatal visits, versus 97.1% and 85.7% in 2011. Only 9.1% had been reimbursed for delivery fee in 2008, but the proportion was raised to 36.8% in 2011. (Table 1).

**Table 1:** Participants’ characteristics.

Characteristics	2008 (before policy adjustment)		2011 (after policy adjustment)		n	%
	n	%	n	%		
*Socio-demographics*						
Age (N = 154)^▲^						
≤ 29 years old	50	64.9	49	63.6	99	64.3
> 29 years old	27	35.1	28	36.4	55	35.7
Education (N = 154)						
≤ Secondary level	45	58.4	37	48.1	82	53.2
> Secondary level	32	41.6	40	51.9	72	46.8
Employment (N = 154)						
Employed	72	93.5	65	84.4	137	89.0
Unemployed	5	6.5	12	15.6	17	11.0
Annual household income (N = 154)^△^						
< 60000 Yuan	46	59.7	25	32.5	71	46.1
≥ 60000 Yuan	31	40.3	52	67.5	83	53.9
*Maternal health care status*						
Prenatal care 5+ (n = 146)						
Yes	69	89.6	67	97.1	136	93.2
No	8	10.4	2	2.9	10	6.8
Postnatal care (N = 154)						
Yes	49	63.6	66	85.7	115	74.7
No	28	36.4	11	14.3	39	25.3
Delivery institution (n = 148)						
Primary institution	48	62.3	28	39.4	76	51.4
Secondary and above	29	37.7	43	60.6	72	48.6
Parity (n = 153)						
Primiparous	61	79.2	58	76.3	119	77.8
Multiparous	16	20.8	18	23.7	34	22.2
Delivery type (n = 151)						
Vaginal	39	50.6	37	50.0	76	50.3
Caesarean	38	49.4	37	50.0	75	49.7
*Reimbursement*						
Reimbursed for childbirth (n = 153)						
Yes	7	9.1	28	36.8	35	22.9
No	70	90.1	48	63.2	118	77.1

^**▲**^the median of age was 29 years old.

^**△**^the median household income was 60000 Yuan ($9117.3).

There were some missing data in this table.

### Maternal services utilization before and after compensation policy adjustment

Multivariate logistic regression analyses revealed more women accepted postnatal visits in 2011 than in 2008 (adjusted OR = 3.32, *p* = 0.009), second-class or above institutions were more likely used for childbirth in 2011 than in 2008 (adjusted OR = 2.32, *p* = 0.03), and there was no significant difference in prenatal care between 2008 and 2011 (*p* = 0.16) (Table 2).

**Table 2:** OR and 95% CI for maternal services utilization.

Characteristics	ORm (95% CI) for model 1	ORm (95% CI) for model 2	ORm (95% CI) for model 3
Before or after compensation scheme adjustment			
Year			
2008	1	1	1
2011	3.41 (0.62,18.81)	3.32 (1.35,8.15)**	2.32 (1.10,4.91)*
Socio-demographics			
Age			
≤ 29 years	1	1	1
> 29 years	1.28 (0.25,6.47)	0.48 (0.19,1.22)	1.40 (0.57,3.40)
Education			
≤ Secondary level	1	1	1
> Secondary level	12.79 (0.53,14.75)	1.50 (0.63,3.57)	3.95 (1.88,8.31)***
Employment			
Employed	1	1	1
Unemployed	2.45 (0.41,14.54)	0.68 (0.16,2.97)	1.25 (0.37,4.32)
Annual household income			
< 60000 Yuan	1	1	1
≥ 60000 Yuan	1.70 (0.37,7.76)	1.80 (0.75,4.32)	1.11 (0.51,2.39)
Maternal health care status			
Parity			
Multiparous	1	1	1
Primiparous	1.71 (0.37,7.76)	2.81 (0.99,7.93)	0.81 (0.29,2.22)

ORm: odds ratio of multivariate logistic analysis; Model 1, 2, and 3: In model 1, the dependant variable was the proportion of prenatal care at least 5 times; in model 2, the dependant variable was proportion of postnatal visits; in model 3, the dependant variable was proportion of delivery in the secondary and above institutions

*p < 0.05

** *p* < 0.01

*** *p* < 0.001.

The chi-square for the Hosmer & Lemeshow test of the three models (model 1, 2, and 3) was 4.75 (*p* = 0.78), 6.56 (*p* = 0.59), and 10.33 (*p* = 0.24), respectively, and the fit of the multivariate logistic regression models were good. The Hosmer-Lemeshow test is a statistical test for goodness of fit for the logistic regression model. A large value of ^2^ with *p* < 0.05 indicates poor fit and a small ^2^ indicates a good logistic regression model fit [16].

### Reimbursement rate before and after compensation policy adjustment

In 2008, only 9.1% of enrolled mothers received reimbursement from the NCMS; however, the rate increased to 36.8% after the NCMS policy adjustment in 2011 (Table 3). A univariate analysis revealed that there was a great improvement in the proportion of mothers who received reimbursement (ORu = 5.83, *p* < 0.001).

**Table 3:** OR and 95% CI for reimbursement.

Characteristics	Frequency	Row%	ORu^1^(95% CI)	ORm^2^ (95% CI)	ORm^3^(95% CI)
Before or after compensation scheme adjustment					
Year (n = 153)					
2008	7	9.1	1	1	1
2011	28	36.8	(2.36,14.43)***	(1.61,11.01)**	(1.74,12.68)**
Socio-demographics					
Age (n = 153)					
≤ 29 years	24	68.6		1	1
> 29 years	11	31.4		1.03 (0.41,2.57)	1.08 (0.35,3.35)
Education (n = 153)					
≤ Secondary level	10	12.3		1	1
> Secondary level	25	34.7		3.56(1.45,8.74)**	(1.52,11.66)**
Employment (n = 153)					
Employed	27	19.9		1	1
Unemployed	8	47.1		0.23(0.06,0.83)*	0.24(0.06,0.97)*
Annual household income (n = 153)					
< 60000 Yuan	10	14.3		1	1
≥ 60000 Yuan	25	30.1		2.42 (0.88,6.63)	1.68 (0.58,4.88)
Maternal health care status					
Parity (n = 152)					
Multiparous	7	21.2			1
Primiparous	28	23.5			0.77 (0.20,2.93)
Delivery institution (n = 148)					
Primary institution	12	15.8			1
Secondary and above	22	30.6			1.31 (0.50,3.39)
Delivery type (n = 150)					
Vaginal delivery	13	17.3			1
Caesarean section	22	29.3			1.96 (0.77,5.04)

1: odds ratio of univariate analysis; 2: odds ratio for the first model adjusted by covariates of socio-demographic factors (age, education, employment, income); 3: odds ratio for the second model adjusted by covariates of socio-demographic factors and maternal healthcare status (parity, delivery institution, delivery type); 147 cases were included in secondary model because of some missing data

*p < 0.05

** *p* < 0.01

*** *p* < 0.001.

The results of the multivariate logistic regression analyses revealed that reimbursement rates showed significant differences between 2008 and 2011 after adjusting for covariates both in regression model 1 (adjusted OR = 4.21, *p* = 0.003) and model 2 (adjusted OR = 4.70, *p* = 0.002) (Table 3). The chi-square for the Hosmer & Lemeshow test of the primary model and secondary model was 3.27 (*p* = 0.92) and 5.89 (*p* = 0.66), respectively, and the fit of the multivariate logistic regression models were good [16].

### OOP proportion before and after compensation policy adjustment

The T test results revealed that the proportion of OPP shared in delivery fee did not decrease significantly (*t* = 0.56, *p* = 0.58), nor did the proportion of OOP shared in household income (*t* = 0.42, *p* = 0.68) (Table 4).

**Table 4:** Comparison of OOP proportion between 2008 and 2011.

OOP proportion	2008 (before policy adjustment)	2011 (after policy adjustment)	t	p
OOP proportion in delivery expense %	55.47 ± 27.5	60.71 ± 21.0	0.56	0.58
OOP proportion in household income %	3.28 ± 2.3	3.78 ± 2.9	0.42	0.68

Since income was the only variance that was significantly different (^2^ = 11.53, *p* = 0.001) before and after policy adjustment, a covariance model adjusting for income was used to predict the changes of the OOP proportion between 2008 and 2011. The results revealed that there were no significant differences on the proportions of OOP shared in delivery fee (*F* = 0.24, *p* = 0.63) and in household income (*F* = 0.46, *p* = 0.50) (Table 5).

**Table 5:** Differences in OOP proportion between 2008 and 2011 by covariance analyses.

OOP proportion	2008 (before policy adjustment)	2011 (after policy adjustment)	F	p
OOP proportion in delivery expense %	55.47 ± 27.5	60.71 ± 21.0	-0.24	0.63
OOP proportion in household income %	3.28 ± 2.3	3.78 ± 2.9	-0.46	0.50

## Discussion

The NCMS has been the focus of China's rural medical security-system construction in recent years. Earlier research indicated medical insurance was related closely to maternal and child health. In the 1980s, medical insurance was internationally started using to promote maternal and child health-service provisions. It was determined that health insurance enhanced the utilization of maternal and child health services and improved the health status of women and children [13, 17–19].

Domestic studies have reported a negative correlation between basic medical insurance rates and maternal mortality, and that maternal mortality rate was lower in districts with a more sound social security system [20]. According to the NHSS in 2003, the institutional delivery rate, as well as the infant survival rate was significantly different between women covered and not covered by the cooperative medical care system [13]. Pregnant women with health insurance accepted more prenatal examinations than those without health insurance [21].

According to the results of this study, the NCMS regulation promotion positively affected maternal services utilization. The rate of postnatal visits rose significantly among participants. This might be because the new policy was more popular than before and it effectively stimulated the willingness of those mothers to seek healthcare services.

Another important finding was that the proportion of delivery in second-class or above institutions had increased among mothers. Mothers’ incentive to seek higher-grade services improved after policy adjustment. With the increased compensation proportion, the services expense was now affordable for poorer residents. This result supports that the new policy played a role in reducing poorer residents’ economic burden concerning maternal healthcare.

However, increasing delivery in higher-level hospitals was not the purpose of the new policy, and this phenomenon might be explained by the rising fee of maternal services. Earlier research found the appropriate reimbursement ratio could effectively shunt the patients to different-level institutions [22]; however, our study yielded no similar results. This indicates the new policy in Yuyao did not work in promoting the grading clinics, and further adjustment strategies should be considered to set the reimbursement proportion more rationally.

This study also revealed that the reimbursement rate could increase with the degree of NCMS compensation increase. Nevertheless, it was still at a low level; this suggests that a certain number of women covered by medical insurance still did not receive compensation for a variety of reasons.

Some relevant domestic studies found that the floating population was one of the important factors influencing NCMS practice. Many of the rural migrant workers were women of childbearing age, and they had trouble receiving compensation for their medical expenses that occurred away from their homeland [23]. Inadequate publicity resulted in less knowledge about NCMS regulations in rural women, which also may have affected the compensation rate [24, 25].

The childbirth expense in Yuyao increased between 2008 and 2011; however, the OOP proportion did not change. The effect of adjusting the compensation regulation did very little to reduce the economic burden of rural hospital childbirth. This suggests that due to some objective reasons such as the rising cost of health care, the government’s goal to reduce the economic burden of maternal healthcare was difficult to achieve in rural areas, even though the compensation scheme was improved. At least for now, the effect was not obvious.

### Study limitations

This study has one significant limitation. The sample only included participants from Yuyao who had experienced childbirth in the last five years with NCMS coverage. Consequently, the association between NCMS policy adjustment on maternal services utilization and expenditures can be discussed with this sample; however, conclusions cannot be generalized to the entire population.

Besides NCMS, the implementation of other public health policies during the study period could also affect the maternal service utilization and expenses, and these potential impacting factors should be considered conjunctively in future studies.

## Conclusion

The NCMS adjustment program in Yuyao raised the reimbursement rate; however, it was not helpful in reducing the economic burden of rural maternal healthcare. The improved rate of postnatal visits indicated that the NCMS policy not only promoted institutional delivery as some recognized findings reported, but also played a role in other maternal care uses. In general, the improved NCMS policy did promote maternal child healthcare development in rural Yuyao; however, this effect was limited. The design of the compensation scheme must be further adjusted in practice.

## Supporting Information

S1 Questionnaire (DOCX)

S1 Dataset (part)
(SAV)
