# Correction: Comparing Maternal Services Utilization and Expense Reimbursement Before and After the Adjustment of the New Rural Cooperative Medical Scheme Policy in Rural China

**DOI:** 10.1371/journal.pone.0170617

**Published:** 2017-01-18

**Authors:** Hua You, Hai Gu, Weiqing Ning, Hua Zhou, Hengjin Dong

An affiliation for the first author was incorrectly omitted. The affiliations should appear as shown here:

Hua You^1,2,3^, Hai Gu^3^, Weiqing Ning^4^, Hua Zhou^5^, Hengjin Dong^1^*

1 Center for Health Policy Studies, School of Medicine, Zhejiang University, Hangzhou, China

2 Department of Social Medicine and Health Education, School of Public Health, Nanjing Medical University, Nanjing, China,

3 Center for Health Management and Care Security Policy Research, School of Government, Nanjing University, Nanjing, China,

4 Department of Women Health Care, the First Affiliated Hospital of Nanjing Medical University, Nanjing, China,

5 Department of Child Health Care, Maternal and Child Health Hospital of Changzhou, Changzhou, Jiangsu, China

## References

[1] You H, Gu H, Ning W, Zhou H, Dong H (2016) Comparing Maternal Services Utilization and Expense Reimbursement before and after the Adjustment of the New Rural Cooperative Medical Scheme Policy in Rural China. PLoS ONE 11(7): e0158473 doi: 10.1371/journal.pone.0158473 27388439 10.1371/journal.pone.0158473 PMC4936705

