# Supplementary material for: Comparing Maternal Services Utilization and Expense Reimbursement before and after the Adjustment of the New Rural Cooperative Medical Scheme Policy in Rural China
Source: PLoS One. 2016 Jul 7;11(7):e0158473. doi: 10.1371/journal.pone.0158473 (PMC4936705; doi:10.1371/journal.pone.0158473)
Supplement: S1 Questionnaire — (DOCX) [file pone.0158473.s001.docx]

**Explanation for the “questionnaire”**

The whole household health questionnaire used in the National Health Service Survey (NHSS) includes 8 instruments and in this study we just used part of them. So, the related questions and answers were listed and translated in English as below:

**家庭一般情况问卷Instrument for Characteristics of The Family:**

您家年收入是多少（元）？（纯收入）

How much was your annual household income(net income)?

您家是否参加了新型农村合作医疗？

Did you take part in the New Rural Cooperative Medical Scheme?

**家庭成员健康调查问卷Instrument for Characteristics of The Family Members:**

出生日期 （年） （月）

Date of birth (year) (month)

婚姻状况 未婚 在婚 离婚 丧偶 其他

Marital status: (1) unmarried (2) currently married (3) divorced (4) widowed  (5)or other

文化程度 没上过学 小学 初中 高中/技校 中专/中技 大专 大学及以上

Educational level: (1)Haven't been to school (2)primary school (3)junior middle school (4)high school/ technical school (5)secondary technical school (6)junior college (7)university and above

就业状况 在业 离退休 在校学生 无业或失业

The employment situation: (1)employed (2) retired  (3)student in the school (4)unemployed

**15-49岁已婚育龄妇女调查表 Instrument for married women of childbearing age(15-49 years old)**

您曾经生了几个孩子（包括现在已经去世的）？

How many children did you deliver?

您在最近3年内是否有分娩 （1）是 （2）否

Did you deliver in recent three years? (1)yes (2)no

最后一个孩子的出生日期： （年） （月）

Date of birth for the last child: (year) (month)

产前做过几次检查（次）？

How many prenatal care services did you accept?

您在哪里做的产前检查？县/区及以上医院 县/区及以上中医院 妇幼保健机构

乡镇街道卫生院 社区卫生服务中心 计划生育指导站 卫生室 其他

What institution did you accept prenatal care in? (1)county-level hospitals (2)county-level Chinese traditional hospitals (3)maternity and child care institution (4)health clinics in towns and townships (5)community health service centers (6)Family planning guidance station (7)Village clinics (8)others

出生方式：顺产 阴道助产 剖腹产

Childbirth way? vaginal delivery  cesarean section

分娩地点：县/区及以上医院 县/区及以上中医院 妇幼保健机构

乡镇街道卫生院 社区卫生服务中心 计划生育指导站 卫生室 家中 其他

Delivery institution? (1)county-level hospitals (2)county-level Chinese traditional hospitals (3)maternity and child care institution (4)health clinics in towns and townships (5)community health service centers (6)Family planning guidance station (7)Village clinics (8) in the home (9)others

分娩费用总共多少（元）？

How much did you pay for the delivery(Yuan)?

分娩费用补偿或报销了多少（元）？(没有填0)

How much did you receive the reimbursement for delivery fee?（filled with “0” if no reimbursement）

产后42天内，您接受产后访视的次数？(没有填0)

How many postnatal visits did you accept in the 42 days after delivery? （filled with “0” if having no postnatal visit）
